# GFPnovo2, a brighter GFP variant for in vivo labeling in *C. elegans*

**DOI:** 10.17912/49YB-7K39

**Published:** 2018-09-06

**Authors:** Ardalan Hendi, Kota Mizumoto

**Affiliations:** 1 Department of Zoology, University of British Columbia, Vancouver Canada

**Figure 1:**
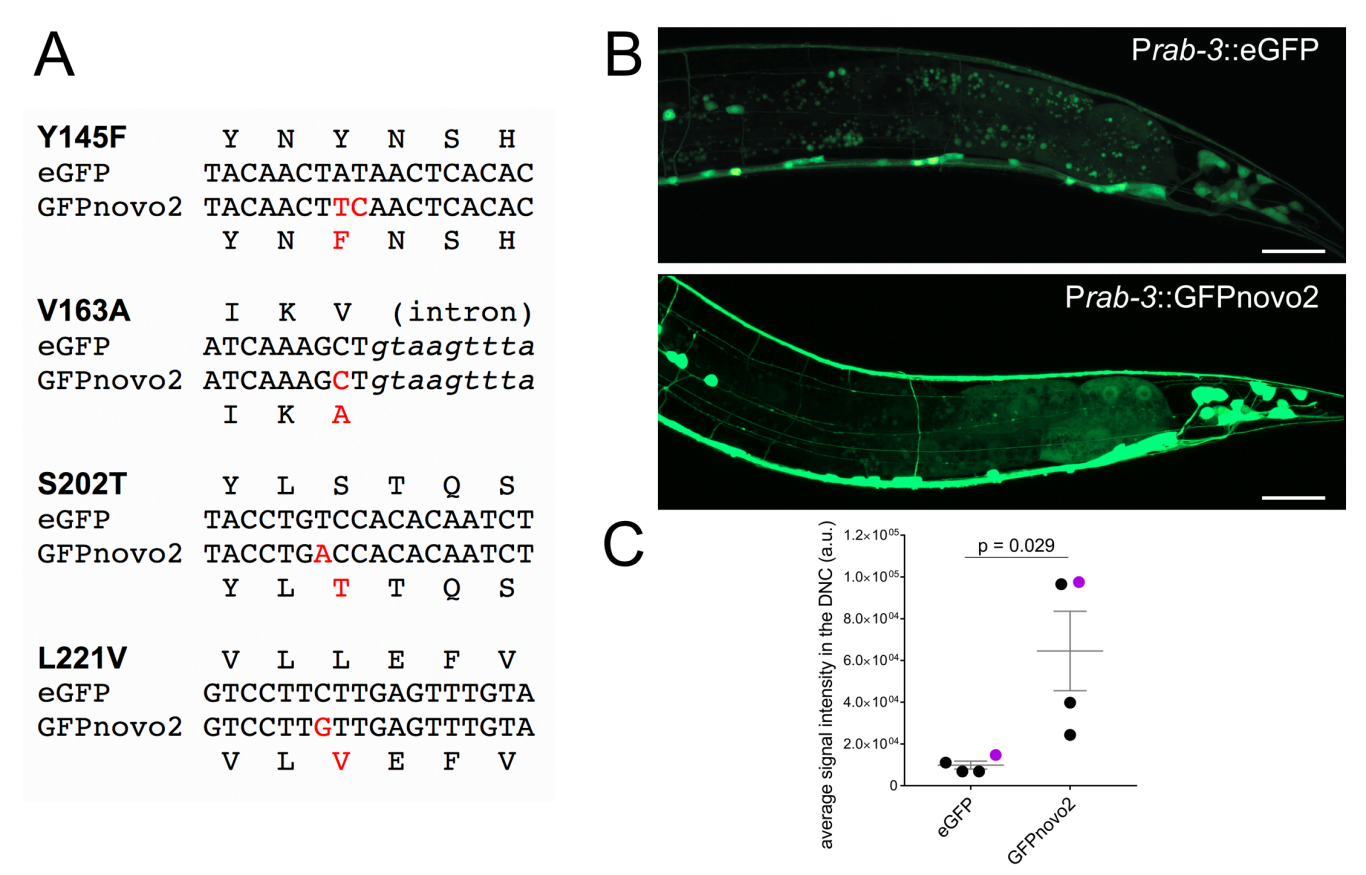
(A) The four mutations introduced in the original eGFP in pSM vector to generate GFPnovo2. The pSM vector contains the codon-optimized eGFP with three synthetic introns. DNA sequence of eGFP (top) and GFPnovo2 (bottom) along with their amino acid sequences are aligned. Red letters indicate mutations in the DNA and corresponding amino acid residues. The complete sequence of the pSM.GFPnovo2 vector is available from Addgene. (B) Representative confocal images of posterior region of adult animals expressing P*rab-3::*eGFP (*mizEx328*) (top) and P*rab-3::*GFPnovo2 (*mizEx329*). The identical imaging setting was used for taking these images. The 488nm laser was used to excite both eGFP and GFPnovo2. Scale bar: 20mm. (C) Comparison of the signal intensity of the dorsal nerve cord. 100mm region of the dorsal nerve cord was used for quantifying the average signal intensities. Each dot represents the individual animal. Purple dots represent animals shown in (B).

## Description

Green fluorescent protein (GFP) is one of the most common fluorophores used to label cells and proteins in *C. elegans*. Previous artificial protein evolution strategy has generated a variant of eGFP, GFPnovo2, which is 3.3 times brighter than the original eGFP in the DT40 cell line (Arakawa et al, 2008). GFPnovo2 carries four mutations from the original eGFP (Y145F, V163A, S202T, L221V), and has the same excitation/emission wavelengths as the original eGFP. Here we compared the brightness of GFPnovo2, which was generated by introducing above mentioned mutations in the pSM (eGFP_unc-54 3’ utr: kind gift from Cori Bargmann) vector (Figure 1A), with that of original eGFP in the *C. elegans* nervous system. Under the fluorescent dissection scope, all four GFPnovo2 lines with high transmission rate (over 80%) we examined had brighter fluorescent signal at all developmental stages (late embryo to adult) than five eGFP lines with the similar high transmission rate. As axons and dendrites are thin, it is often difficult to visualize when the copy number of the transgene is low. Indeed, we observed fewer neurites when we labeled neurons with the low dose of eGFP (1ng/ml) expressed under the pan-neuronal promoter, P*rab-3* (Figure 1B, top). In contrast, GFPnovo2 was considerably brighter than eGFP and nicely labeled neurites when injected at the same concentration (1ng/ml) (Figure 1B, bottom). As a result, the average signal intensities of the dorsal nerve cord, which contains only neurites, was significantly and consistently brighter in the animals expressing GFPnovo2 than those expressing eGFP (Figure 1C). The variation among the animals expressing GFPnovo2 is likely due to the mosaic nature of the extra-chromosomal array. Nevertheless, all GFPnovo2 animals had a brighter signal than eGFP animals. Similarly, we were able to label the entire axon of DA9 neuron with GFPnovo2 expressed under the DA9 specific promoter, *itr-1* (Chen et al., 2018), including the axonal tip which was not easy to detect with eGFP (unpublished). We did not notice detectable photobleaching while examining animals expressing GFPnovo2 under the fluorescent compound microscope or the confocal microscope, suggesting that GFPnovo2 is at least as stable as eGFP.

**Qualifiers**

1. In this manuscript, we did not conduct definitive comparison between GFPnovo2 and eGFP using single-copy integration of the transgene. The comparison is therefore rather subjective, even though the difference is very noticeable at the dissection microscopy level.
2. We have not tested whether the reagents for GFP such as antibodies and GFP nanobody::ZIF-1 for tissue specific protein degradation (Wang et al., 2017) could be used with GFPnovo2. Arakawa et al. suggested that the overall structures of GFPnovo2 is unlikely to be changed much.
3. We have so far tested GFPnovo2 only in the nervous system (the leaky expression of the P*rab-3* and *Pitr-1* suggests GFPnovo2 is also brighter in the hindgut). It is therefore not known if GFPnovo2 is brighter in all tissues at any given developmental stage.

**Conclusion**

GFPnovo2 could be the useful option for labeling cells and proteins in *C. elegans*, especially when the copy number of the transgene is low such as single-copy integration or CRISPR-mediated tagging of the endogenous proteins.

## Reagents

Plasmid: pSM.GFPnovo2 Addgene #116943

Strains: UJ1000: mizEx328 [Prab-3::eGFP;Podr-1::GFP]; UJ1001: mizEx329 [Prab-3::GFPnovo2;Podr-1::GFP].
